# Conditional random slope: A new approach for estimating individual child growth velocity in epidemiological research

**DOI:** 10.1002/ajhb.23009

**Published:** 2017-04-21

**Authors:** Michael Leung, Diego G. Bassani, Amy Racine‐Poon, Anna Goldenberg, Syed Asad Ali, Gagandeep Kang, Prasanna S. Premkumar, Daniel E. Roth

**Affiliations:** ^1^ Research Institute and Centre for Global Child Health, Peter Gilgan Centre for Research and Learning, The Hospital for Sick Children Toronto Canada; ^2^ Dalla Lana School of Public Health University of Toronto Toronto Canada; ^3^ Department of Paediatrics The Hospital for Sick Children and University of Toronto Toronto Canada; ^4^ Novartis Pharma AG Basel Switzerland; ^5^ Genetics and Genome Biology, Peter Gilgan Centre for Research and Learning, The Hospital for Sick Children Toronto Canada; ^6^ Department of Pediatrics The Aga Khan University Karachi Pakistan; ^7^ Department of Gastrointestinal Sciences Christian Medical College Vellore India; ^8^ Department of Biostatistics Christian Medical College Vellore India

## Abstract

**Objectives:**

Conditioning child growth measures on baseline accounts for regression to the mean (RTM). Here, we present the “conditional random slope” (CRS) model, based on a linear‐mixed effects model that incorporates a baseline‐time interaction term that can accommodate multiple data points for a child while also directly accounting for RTM.

**METHODS:**

In two birth cohorts, we applied five approaches to estimate child growth velocities from 0 to 12 months to assess the effect of increasing data density (number of measures per child) on the magnitude of RTM of unconditional estimates, and the correlation and concordance between the CRS and four alternative metrics. Further, we demonstrated the differential effect of the choice of velocity metric on the magnitude of the association between infant growth and stunting at 2 years.

**RESULTS:**

RTM was minimally attenuated by increasing data density for unconditional growth modeling approaches. CRS and classical conditional models gave nearly identical estimates with two measures per child. Compared to the CRS estimates, unconditional metrics had moderate correlation (*r* = 0.65–0.91), but poor agreement in the classification of infants with relatively slow growth (kappa = 0.38–0.78). Estimates of the velocity‐stunting association were the same for CRS and classical conditional models but differed substantially between conditional versus unconditional metrics.

**CONCLUSION:**

The CRS can leverage the flexibility of linear mixed models while addressing RTM in longitudinal analyses.

## 1 INTRODUCTION

Estimation of the rate of change over time in a child's physical size is essential for epidemiological studies of the determinants and consequences of variations in child growth. Methods to estimate growth velocity in early life are particularly relevant to the developmental origins of health and disease (DOHaD) hypothesis; for example, the pace of early postnatal growth or weight gain may influence the risk of obesity and cardiometabolic diseases later in life (Victora et al., 2008). However, there is currently a lack of consensus on the operational definition of growth velocity, leading to inconsistent use of statistical strategies to quantify growth in epidemiology.

Estimates of growth velocity within a particular age interval should ideally be independent of the child's size at the beginning of the interval (Cole, 1995, 1998; Keijzer‐Veen et al., 2005). Uncoupling growth from baseline size enables inferences about growth that are specific to a particular developmental window, by removing the effect of regression to the mean (RTM) which is the natural tendency of extreme measures to be closer to the population mean size on a subsequent or preceding time point (Cameron, Preece, & Cole, 2005; Cole, 1994). The phenomenon of RTM is particularly relevant in the first few years of life when there is substantial inter‐individual variability in growth patterns, before children settle into stable size rankings with respect to peers (Cole, 1995; Mei, Grummer‐Strawn, Thompson, & Dietz, 2004).

Conditional growth metrics have been applied in numerous DOHaD studies of the association between early linear growth and chronic disease risk factors later in life (Adair et al., 2013; Keijzer‐Veen et al., 2005). However, the classical formulation of conditional velocity relies on only two measurement time points, and therefore does not accommodate multiple anthropometric measurements for each child within the interval. Flexible methods for handling longitudinal data have been applied to model early childhood growth trajectories, such as linear mixed‐effects (LME) models using linear spline functions (Howe et al., 2013) and nonlinear approaches such as SuperImposition by Translation and Rotation (SITAR) (Beath, 2007; Cole, Donaldson, & Ben‐Shlomo, 2010; Johnson, van Jaarsveld, Llewellyn, Cole, & Wardle, 2014; Jones‐Smith et al., 2013); however, these approaches are typically “unconditional” in that they do not directly account for RTM. Increasing the data density (i.e., number of observations per child within a specified age interval) could indirectly minimize the effect of RTM if mid‐interval data sufficiently overcome the leverage of the baseline value; alternatively, the estimation of individual‐specific random slopes (i.e., best linear unbiased predictors, or BLUPs) may account for RTM, as the estimate of velocity is shrunk towards the group mean relative to the observed values (Robinson, 1991). However, neither increasing data density nor the performance of BLUPs have been empirically tested with respect to their effects on RTM in the context of infant growth analyses.

Here, we propose the “conditional random slope” (CRS), a hybrid longitudinal and conditional approach based on a LME model that is a direct generalization of the classical conditional growth approach, but can leverage the availability of multiple and variable timed data points within the age interval of interest. In this study, we focused on linear growth from 0 to 12 months because of the considerable inter‐individual variability in linear growth in this age range (leading to substantial RTM), and because it is a period of public health relevance in which growth‐sensitive interventions are most likely to have long‐term effects (Stein et al., 2010; Victora, de Onis, Hallal, Blossner, & Shrimpton, 2010). The specific objectives of this study were to: (1) determine whether the effect of RTM on unconditional estimates of growth velocity is attenuated by increasing the density of individual‐level observations from 0 to 12 months; (2) evaluate the correlation and agreement in classification between the CRS and four selected alternative conditional and unconditional growth velocity metrics; and (3) compare the different infant velocity metrics in terms of the magnitude of their associations and predictive accuracies for stunting at 2 years.

## 2 METHODS

### 2.1 Data sources

We used two datasets that were accessed through the Healthy Birth, Growth, and Development Knowledge Integration (HBGDki) program funded by the Bill and Melinda Gates Foundation (Table 1). Cohorts A and B were birth cohorts from Vellore, India (Paul, Gladstone, Mukhopadhya, & Kang, 2014), and Karachi, Pakistan (Global Grand Challenges, 2012), respectively.

**Table 1:** Description of data sources

	Cohort A	Cohort B
Years of enrolment	2002–2006	2012–2014
Country	India	Pakistan
Age at observation	Birth to 3 years	Birth to 2 years
Sample recruited	373	380
Excluded due to incomplete data^a^, *n* (%)	25 (6.7%)	18 (4.7%)
Revised sample size	348	362
Number of observations from birth to 12 months of age per child, median (range)	11 (5, 13)	12 (6, 13)
Age in days at baseline observation,^b^ median (range)	29 (1, 66)	7 (1, 68)
Baseline LAZ, mean ± SD	−0.94 ± 1.3	−1.41 ± 1.7
Age in days at follow‐up, median (range)	347 (316, 365)	340 (306, 355)
Follow‐up LAZ, mean ± SD	−1.97 ± 1.3	−2.64 ± 1.1

a Children with fewer than 5 observations from birth to 12 months were excluded from all analyses.

b Day 1 is day of birth.

Length measurements were scheduled monthly in both cohorts, yet actual frequencies and timings of anthropometric measurements differed across the datasets (Table 1). To generate comparable estimates of growth velocity, observations were selected so that each child had the same individual‐level data density from 0 to 12 months; for example, for a data density of three observations per child, only the closest observations to 0, 6, and 12 months were retained in the analysis for each child (details on sampling of observations can be found in Supporting Information Table A1). To ensure that the same children were included in all analyses irrespective of data density, those with fewer than five observations from 0 to 12 months were excluded. We performed sensitivity analyses in which we excluded children for whom the earliest observation occurred beyond the first week of life.

We modeled length‐for‐age *z*‐score (LAZ) using the World Health Organization (WHO) Child Growth Standards (WHO Health Organization, 2006) instead of raw length for the following reasons: (1) whereas the mean trajectory of raw length is a nonlinear function of age, the null hypothesis is that the mean LAZ trajectory is linear in all intervals, thereby simplifying the model; and, (2) mean LAZ velocity of the study population can be compared to the standard normal pattern for healthy children. We excluded extreme LAZ values based on WHO recommendations (LAZ > 6 or LAZ<−6).

To allow for comparisons across metrics within each cohort, we internally standardized the velocity estimates for the cohort‐specific distribution of observed velocities within the interval (i.e., divided each child's velocity estimate by the empirical standard deviation of the velocities across the cohort). For each metric, the standardized velocity is a velocity *z*‐score (analogous to LAZ), whereby each child's estimate can be interpreted as a relative measure of his/her growth rate compared to the mean of the cohort. In this article we focus on growth in length, but the principles can be applied to other anthropometric variables.

### 2.2 Velocity metrics

We estimated cohort‐average growth trajectories, and individual growth velocities for the analyses using the CRS approach, a classical approach to conditional growth, and three unconditional growth velocity metrics:

#### 2.2.1 Unconditional change in LAZ

$$\Delta LAZ_{j}\;=\;LAZ_{1j}\;–\;LAZ_{0j}$$

LAZ_ij_ is LAZ at age *i* for child *j*. ΔLAZ_j_ is the arithmetic difference between LAZ at the end (LAZ_1j_) and beginning (LAZ_0j_) of the interval of interest. This unconditional difference can be considered a metric of velocity when the interval duration is constant for all children.

#### 2.2.2 Classical conditional change in LAZ

$$LAZ_{1j}=\;\beta_{0}+\beta_{1}(LAZ_{0j})\;+{ɛ}_{ij}$$

The conditional delta LAZ (also referred to as the conditional standard deviation score (SDS)) is the difference between observed and expected LAZ at the end of the interval, where expected value is based on LAZ_0j_. The conditional SDS can be derived by calculating ɛ_ij_, the residual at age *i* for child *j*, after regressing size at or near the end of the interval (LAZ_1j_) on baseline size (LAZ_0j_). As such, ɛ_ij_, represents the portion of LAZ_1j_ that is uncorrelated with LAZ_0j_.

#### 2.2.3 Fixed slope estimate of the change in LAZ (unconditional)

$$LAZ_{ij}=\;\beta_{0j}+\beta_{1j}(t)\;+{ɛ}_{ij}$$

The fixed slope is the individual‐specific slope from a separate linear regression of LAZ on time for each child, whereby β_1j_ represents the rate of change of LAZ_j_ over the specified interval for the infant. This model is a generalization of the unconditional change model, whereby the estimation of velocity is not restricted to using LAZ at only two time points (i.e., beginning and end of the interval of interest).

#### 2.2.4 Random slope estimate of the change in LAZ (unconditional)

$$LAZ_{ij}=\;\beta_{0}+\beta_{1}\left(t_{c}\right)+\mu_{0j}+\mu_{1j}(t_{c})\;+{ɛ}_{ij}$$

$$\left(\begin{array}{c} \mu_{0j}\\ \mu_{1j} \end{array}\right)\;∼\;N\;\left\{\left(\begin{array}{c} 0\\ 0 \end{array}\right),\;\left(\begin{array}{cc} \sigma_{\mu 0j}^{2} & 0\\ 0 & \sigma_{\mu 1j}^{2} \end{array}\right)\right\}$$

$${ɛ}_{ij}∼\;N(0,\sigma_{ɛ}^{2})$$

The random slope is the child‐specific rate of change in LAZ_j_ relative to the group mean over a specified interval, where LAZ_ij_ is LAZ at age *i* for subject *j*; β_0_ and β_1_ are fixed effects; μ_0j_ and μ_1j_ are subject‐specific random effects, *t*_c_ is centered age and ɛ_ij_ is the residual error. The random effects are assumed to follow a bivariate normal distribution with zero means with variances $\sigma_{\mu 0}^{2}$ and $\sigma_{\mu 1}^{2}$; and ɛ_ij_ are assumed to follow a normal distribution with mean zero and variance $\sigma_{ɛ}^{2}$. An unstructured covariance matrix is typically selected for growth models to allow variance and covariance estimates to be distinct (Johnson, Balakrishna, & Griffiths, 2013); however, due to issues with model convergence at low data density, we used an independent covariance matrix instead with the age variable centered, whereby the random effects may have unique variances but do not co‐vary. This is a reasonable assumption since the slope is theoretically uncorrelated with the age‐centered intercept (mean LAZ over the specified interval) (Blance, Tu, & Gilthorpe, 2005; Oldham, 1962). However, we used an unstructured covariance matrix in a sensitivity analysis. Covariance of within‐subject residuals was assumed to be zero; however, inferences were unchanged in models that used first‐order autoregressive residual correlation matrices (not shown).

#### 2.2.5 Conditional random slope estimate of the change in LAZ

$$LAZ_{ij}=\;\beta_{0}+\beta_{1}\left(t_{c}\right)+\mu_{0j}+\mu_{1j}\left(t_{c}\right)+\beta_{2}(t_{c}\times cLAZ_{0j})+{ɛ}_{ij}$$

$$\left(\begin{array}{c} \mu_{0j}\\ \mu_{1j} \end{array}\right)\;∼\;N\;\left\{\left(\begin{array}{c} 0\\ 0 \end{array}\right),\;\left(\begin{array}{cc} \sigma_{\mu 0j}^{2} & 0\\ 0 & \sigma_{\mu 1j}^{2} \end{array}\right)\right\}$$

$${ɛ}_{ij}\;∼\;N(0,\sigma_{ɛ}^{2})$$

Our proposed metric, the conditional random slope (CRS), is based on a similar model as the LME model described above, but with an adjustment for baseline LAZ‐time interaction. LAZ_ij_ is the LAZ at age *i* for subject *j*; β_0_ and β_1_ are fixed effects; μ_0j_ and μ_1j_ are subject‐specific random effects; β_2_ is the coefficient for the baseline LAZ‐time interaction term; cLAZ_0j_ is baseline LAZ centered at the mean; *t*_c_ is centered age and ɛ_ij_ is the residual error. The random effects are assumed to follow a bivariate normal distribution with zero means with variances $\sigma_{\mu 0}^{2}$ and $\sigma_{\mu 1}^{2}$; and ɛ_ij_ are assumed to follow a normal distribution with mean zero and variance $\sigma_{ɛ}^{2}$. The CRS model also assumes an independent covariance structure for the random effects. The independence assumption is identical to that implied by the classical formulation of the conditional SDS (Cole, 1994, 1995, 1998; Keijzer‐Veen et al., 2005) (Supporting Information B). In primary analyses, we did not include the main effect of baseline size (LAZ_0j_) in the model because the variability in baseline LAZ is already captured by the random intercept ( μ_0j_); however, we included a main effect for LAZ_0j_ in a sensitivity analysis.

### 2.3 Analysis

#### 2.3.1 Effect of individual‐level data density on regression to the mean

The degree of RTM was examined by calculating Pearson's correlation coefficients between velocity estimates and baseline size at varying data densities for each method and cohort. Negative velocity‐baseline correlations are indicative of RTM.

#### 2.3.2 Correlation and concordance among growth velocity metrics

Correlations and agreement between the CRS and the four alternative velocity metrics were assessed using Pearson's correlation coefficient, Cohen's Kappa statistic and % discordance with respect to the classification of children as “abnormal,” whereby abnormal was defined as velocity estimates below the metric‐specific 10th percentile. Goodness‐of‐fit for mixed‐effects models was assessed using the Akaike information criterion (AIC) and the Bayesian information criterion (BIC).

#### 2.3.3 Velocity‐stunting association and prediction

To demonstrate the applicability of the CRS in an epidemiological context, we estimated the association between growth velocity in infancy and stunting (LAZ<−2) at 2 years (or latest available age up to 2 years) for each velocity metric and across varying data density. This was a two‐step approach described previously (Anderson et al., 2013), where internally standardized velocity estimates were first estimated by each method and then exported for use as the independent variable in subsequent logistic regression models. Metric‐specific odds ratios (ORs) were presented for decreasing velocity for ease of interpretability; that is, the relative odds of stunting at 2 years for every one standard deviation reduction in velocity from 0 to 12 months. The C‐statistic, which is the area under the curve (AUC) of the receiving operating characteristic (ROC) curve, was also calculated for each model to assess a metric's predictive accuracy for stunting at 2 years.

Analyses and visualizations were performed using STATA version 13 (Stata Corporation, College Station, TX). The Research Ethics Board at the Hospital for Sick Children (Canada) approved the analyses of the anonymized datasets. Data collection for the original cohort studies A and B were approved by The Aga Khan University (Pakistan) and Christian Medical College (India), respectively.

## 3 RESULTS

### 3.1 Study populations

Children with fewer than five observations from 0 to 12 months were excluded, leaving 710 children eligible for the analyses from both cohorts (Table 1). The two cohorts differed in terms of: (1) number of observations per child; (2) baseline and follow‐up LAZ; and (3) age at baseline and follow‐up measurements. Furthermore, population‐average LAZ trajectories of the two cohorts showed varying degrees of deviation from the international standard, but estimates were similar across the different modeling approaches (Supporting Information Table C1). The group mean LAZ slope was determined by visual inspection to be approximately linear in both cohorts (Supporting Information Figure D1).

### 3.2 Effect of data density on regression to the mean

Using unconditional growth modeling approaches, RTM was minimally attenuated by increasing data density in both cohorts (Table 2). Even after incorporating all available data points, there remained a moderate inverse correlation between baseline size and the subsequent rate of change (Table 2).

**Table 2:** Correlation between estimated length‐for‐age *z*‐score (LAZ) velocity from birth to 12 months of age and baseline LAZ using different analytical approaches and variable number of observations per child

**Cohort**	Velocity—Baseline Correlation^a^				
No. of observations per child^b^	Conditional delta vs. baseline	Unconditional delta vs. baseline	Fixed slope vs. baseline	Random slope vs. baseline	Conditional random slope vs. baseline
**Cohort A**					
2	0.00	−0.57	−0.57	−0.57	0.00
3	–	–	−0.56	−0.56	0.00
4	–	–	−0.49	−0.49	0.00
5	–	–	−0.48	−0.48	0.00
All	–	–	−0.41	−0.41	0.00
**Cohort B**					
2	0.00	−0.76	−0.76	−0.76	0.00
3	–	–	−0.76	−0.76	0.00
4	–	–	−0.71	−0.71	0.00
5	–	–	−0.71	−0.71	0.00
All	–	–	−0.62	−0.62	0.00

a All correlation coefficients for unconditional metrics were statistically significant (*P* < 0.05).

b Every child has at least five observations: at or close to birth, at or close to 12 months of age, and up to 3 additional observations included within the interval from birth to 12 months of age. Analyses of “all” observations included the core five and all other available observations to a maximum of 13 observations per child.

### 3.3 Correlation and concordance among growth velocity metrics

**Figure 1:**
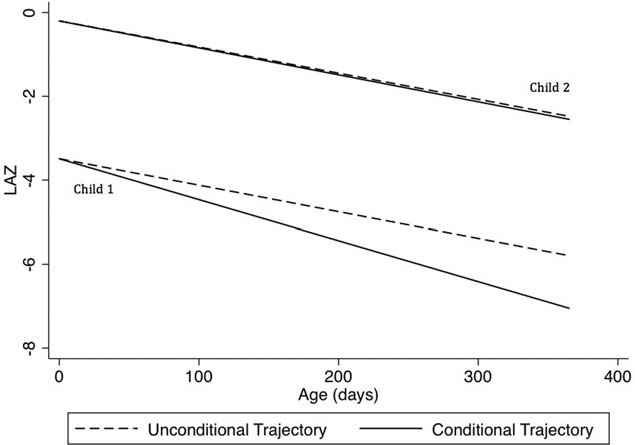
Illustrative examples of the unconditional and conditional trajectories from birth to 12 months of age for two infants from Cohort A. The infants had different baseline length‐for‐age *z*‐scores (LAZ) but similarly declining unconditional growth trajectories (dashed lines). Child 1 had a lower baseline LAZ that was below the group mean and further from the mean compared to Child 2. Therefore, Child 1 had a steeper negative conditional trajectory, implying slower conditional relative growth compared to Child 2. Note that conditioning the change in LAZ on baseline LAZ alters the LAZ scale on which conditional velocities are quantified; therefore, for the conditional trajectories, the predicted LAZ (y‐axis value) that corresponds to a given age (on the *x* axis) cannot be inferred from the conditional trajectories shown in the figure. However, conditional trajectories are plotted to demonstrate conceptually how inferences about growth velocity (i.e., slopes) may differ between unconditional and conditional approaches

Two‐point estimates of the CRS and the conditional delta LAZ were nearly equivalent in both datasets, providing an empirical proof‐of‐concept of the exchangeability of these methods when only two data points per child are available (consistent with the mathematical proof in Supporting Information B). There were strong correlations and agreement among the three unconditional metrics (Supporting Information Tables E1–3). Unconditional metrics had only moderate correlation and poor agreement with the conditional estimates, in both cohorts (Table 3; Figure 1). As expected, these discrepancies were accentuated at the extremes of the velocity distributions, as indicated by the high rate of discordance in the identification of ‘abnormal’ growth in the face of reasonably robust correlation. Furthermore, there was no clear or consistent pattern of improvement or deterioration in correlation/agreement with increasing data density in both cohorts (Table 3). The AICs and BICs for unconditional and conditional random slope models were very similar in both cohorts (Supporting Information Table F1).

**Table 3:** Pairwise comparisons using Pearson's correlation (*R*), Kappa coefficient ( κ), and % discordance (%*D*) between conditional random slopes (CRS) and alternative metrics of growth velocity from birth to 12 months of age, by number of observations per child

**Cohort**	Conditional delta vs.conditional random slope			Unconditional delta vs. conditional random slope			Fixed slope vs. conditional random slope			Random slope vs. conditional random slope		
No. of observations per child^a^	*R* b	κ b, c	%*D* b, c	*R* b	κ b, c	%*D* b, c	*R* b	κ b, c	%*D* b, c	*R* b	κ b, c	%*D* b, c
**Cohort A**												
2	0.99	0.97	0.6%	0.82	0.62	6.9%	0.82	0.65	6.3%	0.82	0.62	6.9%
3	–	–	–	–	–	–	0.82	0.65	6.3%	0.82	0.65	6.3%
4	–	–	–	–	–	–	0.87	0.65	6.3%	0.87	0.62	6.9%
5	–	–	–	–	–	–	0.88	0.78	4.0%	0.88	0.78	4.0%
All	–	–	–	–	–	–	0.91	0.65	6.3%	0.91	0.71	5.2%
**Cohort B**												
2	0.99	1.00	0.0%	0.65	0.44	9.9%	0.65	0.41	10.5%	0.65	0.44	9.9%
3	–	–	–	–	–	–	0.65	0.38	11.1%	0.65	0.38	11.1%
4	–	–	–	–	–	–	0.70	0.51	8.8%	0.70	0.51	8.8%
5	–	–	–	–	–	–	0.70	0.41	10.5%	0.70	0.41	10.5%
All	–	–	–	–	–	–	0.78	0.41	10.5%	0.78	0.48	9.4%

a Every child has at least five observations: at or close to birth, at or close to 12 months of age, and up to 3 additional observations included within the interval from birth to 12 months of age. Analyses of “all” observations included the core five and all other available observations to a maximum of 13 observations per child.

b All correlation and kappa coefficients were statistically significant (*P* < 0.05).

c Kappa and % discordance with respect to the classification of children as “abnormal,” whereby abnormal for each metric was defined as a velocity estimate below the metric‐specific 10th percentile.

### 3.4 Growth velocity in infancy and stunting at age 2 years

**Figure 2:**
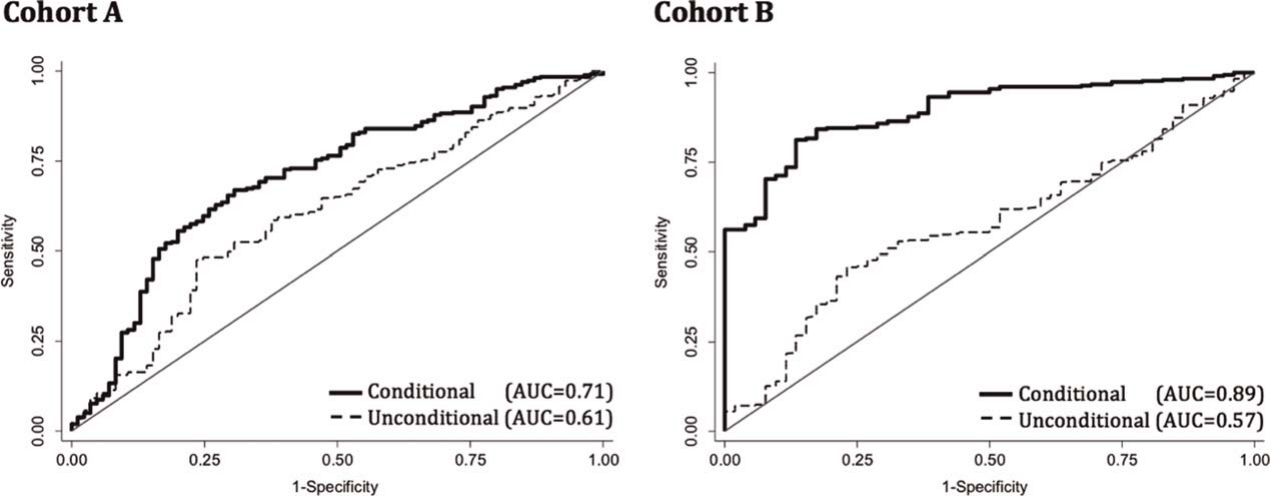
Receiver operating characteristic (ROC) curves for stunting prediction at 2 years of age using two‐point unconditional random slope models and two‐point conditional random slope models. C‐statistics of the individual metrics (equivalent to the area under the curve [AUC] of the ROC curve) are a standard measure of predictive accuracy, where 1.0 would indicate the metric perfectly identifies stunting at 2 years of age, and 0.5 would imply the metric is no better than chance

The expected association between slower growth velocity in infancy and higher odds of stunting at or near 2 years was very similar between CRS estimates versus conditional SDS in the two‐point models; however, increasing the number of observations per child progressively attenuated the estimates based on the CRS models (Table 4). Associations with stunting were consistently stronger for conditional velocity metrics, irrespective of data density, in comparison to unconditional velocity metrics (Table 4; Figure 2).

**Table 4:** Comparison of the strength of association between stunting at 2 years of age and internally standardized estimates of growth velocity, using different analytical approaches and varying number of observations per child

**Cohort**	Conditional delta		Unconditional delta		Fixed slope		Random slope		Conditional random slope	
No. of observations per child^a^	OR^b^ (95% CI)	C^c^	OR^b^ (95% CI)	C^c^	OR^b^ (95% CI)	C^c^	OR^b^ (95% CI)	C^c^	OR^b^ (95% CI)	C^c^
**Cohort A**										
2	2.19 (1.64, 2.91)	0.71	1.46 (1.14, 1.88)	0.61	1.47 (1.14, 1.89)	0.61	1.45 (1.13, 1.86)	0.61	2.11 (1.60, 2.80)	0.71
3	–	–	–	–	1.47 (1.14, 1.89)	0.61	1.45 (1.13, 1.87)	0.61	2.11 (1.59, 2.79)	0.71
4	–	–	–	–	1.46 (1.14, 1.88)	0.60	1.46 (1.14, 1.87)	0.60	1.95 (1.48, 2.57)	0.68
5	–	–	–	–	1.56 (1.22, 2.02)	0.62	1.56 (1.21, 2.00)	0.62	2.10 (1.59, 2.78)	0.70
All	–	–	–	–	1.58 (1.23, 2.03)	0.63	1.57 (1.22, 2.02)	0.63	2.00 (1.52, 2.63)	0.69
**Cohort B**										
2	8.76 (4.90, 15.69)	0.89	1.30 (0.96, 1.76)	0.57	1.30 (0.96, 1.75)	0.57	1.32 (0.97, 1.79)	0.58	9.03 (5.01, 16.3)	0.89
3	–	–	–	–	1.30 (0.96, 1.75)	0.57	1.31 (0.97, 1.77)	0.57	9.39 (5.16, 17.07)	0.90
4	–	–	–	–	1.37 (1.02, 1.84)	0.59	1.38 (1.02, 1.85)	0.59	6.10 (3.74, 9.95)	0.87
5	–	–	–	–	1.34 (0.99, 1.80)	0.59	1.35 (1.00, 1.81)	0.59	5.71 (3.55, 9.21)	0.87
All	–	–	–	–	1.37 (1.02, 1.83)	0.60	1.38 (1.03, 1.85)	0.60	3.38 (2.32, 4.92)	0.81

a Every child has at least five observations: at or close to birth, at or close to 12 months of age, and up to three additional observations included within the interval from birth to 12 months of age. Analyses of “all” observations included the core five and all other available observations to a maximum of 13 observations per child.

b Odds of stunting at 2 years of age for every 1 SD decrease in internally standardized estimates of growth velocity.

c C‐statistic: area under the receiving operating characteristic (ROC) curve.

### 3.5 Sensitivity analyses

Inferences from the following three sensitivity analyses were the same as primary analyses: (1) using an unstructured covariance structure for the unconditional random slope model to demonstrate that unstructured versus independent covariance yielded similar inferences (Supporting Information Tables G1–3); (2) including baseline as a main effect in the CRS approach in order to demonstrate that the inferences were not quantitatively affected by its exclusion in primary analyses (Supporting Information Tables H1–3); and (3) excluding children with a baseline measurement beyond the first week of life to limit the potential error in the estimation of the interaction term of the CRS approach (Supporting Information Tables I1–3).

## 4 DISCUSSION

The CRS is a novel approach for estimating conditional growth velocity based on a LME model that incorporates a baseline size‐age interaction term. This model is straightforward, leverages the flexibility of a longitudinal mixed model while addressing RTM, and is a generalization of the classical formulation of the conditional SDS model (Cole, 1995, 1998; Keijzer‐Veen et al., 2005). We demonstrated that when more than two measurements per child are available for an age interval of interest, CRS models provide a feasible approach for quantifying inter‐individual variations in child growth while accounting for RTM.

This study demonstrated the parallels between the CRS and classical SDS models as well as drew important distinctions between unconditional and conditional growth metrics. First, we showed that the conditional approach could not be simply approximated by increasing the number of intra‐interval data points or estimation by random effects in unconditional models. Increasing the number of individual‐level observations did not attenuate RTM, as there was substantial leverage by baseline and follow‐up sizes irrespective of the addition of mid‐interval values. Furthermore, the use of BLUPs alone (unconditional random slope model) did not eliminate RTM. Even though a child's estimates were shrunk toward the mean trajectory, the degree of shrinkage was proportional across the cohort (due to the use of balanced data), such that children's relative rankings remained the same and the coupling of baseline to slope still remained. Therefore, irrespective of data density, conditional and unconditional metrics were discordant with respect to the identification of children with relatively low growth velocity. In some instances, approximately half of the children experiencing “abnormal” growth by conditional methods were classified as “normal” using unconditional velocity measures. Although we defined growth as abnormal only at the lower end of the distribution (i.e., velocity at or below the 10th percentile), the phenomenon is symmetrical and would be analogous for the “fastest growing” 10% of children.

Unconditional models also yielded estimates of the velocity‐stunting association that were attenuated relative to conditional metrics; this was most clearly demonstrated in Cohort B, in which two‐point unconditional velocity estimates were not associated with stunting at 2 years, whereas the conditional metrics yielded robust associations and high predictive accuracy. The reason for this discordance is that stunting at 2 years is highly correlated with LAZ at 1 year (LAZ_1j_), which is algebraically more similar to conditional velocity (LAZ_1j_‐r*LAZ_0j_) than unconditional velocity (LAZ_1j_ – LAZ_0j_) given that *r* < 1 (i.e., there is imperfect correlation between LAZ at baseline and LAZ at 1 year of age). The purpose of this analysis was to demonstrate the differential effect of the choice of velocity metric in an epidemiological context. However, the relative strengths of associations of conditional versus unconditional velocity metrics would be expected to differ for other outcomes that are less directly related to LAZ (e.g., obesity, neurodevelopment).

The most important contribution of this study was the empirical and algebraic demonstration that the CRS provides a general solution to the classical conditional SDS (Cole, 1995, 1998). Estimates of individual‐level velocity were nearly perfectly correlated and concordant between the two‐point CRS and classical SDS models, and the magnitude of the association of infant growth velocity with stunting at 2 years of age was also nearly identical. However, the approaches differ in that the conditional SDS relies only on data at the beginning and end of the age interval that must be observed for every child and therefore mid‐interval measurements are ignored. Longitudinal data structure may be a disincentive to using the classical formulation, as data within an interval of interest are often dense (>2 observations per individual) and flexible (variable frequency and timing of observations). An additional advantage of the CRS method over the classical conditional approach is that the population‐average slope is estimated in the same model as the child‐specific random slopes, when both baseline LAZ and age are centered at their respective means (Supporting Information Table C1). Therefore, each child's velocity can be reported as a deviation from the international standard by summing the group‐specific fixed effect and child‐specific random effect, while maintaining appropriate correction for child‐level regression to the empirical group mean. In most epidemiological analyses, the focus will be on inter‐individual variation that is entirely captured by the child‐specific random effects alone because the fixed effect is constant across all individuals. However, for disadvantaged populations in countries with high burdens of child growth faltering, it is important to be able to describe both the child's deviation from the group mean and the group's deviation from the international standard.

Despite its theoretical appeal, the practical advantages of the CRS remain to be explored. For example, we found that both the CRS approach and the conditional SDS seem to allow for variable timing of baseline size, as we used two datasets that differed with regards to the timing of baseline LAZ. Although the variable timing of baseline measurement may bias the estimation of the interaction term for the CRS approach (or the correlation coefficient between LAZ_1_ and LAZ_0_ for the conditional SDS), inferences from the main analyses were the same between and within datasets when analyses were restricted to infants with baseline measures in the first week of life (Supporting Information Tables I1–3), perhaps because the range of ages for which baseline occurred was a minor fraction of the total interval from 0 to 12 months. Future work should assess the appropriate timeframe for the consideration of a baseline measurement, as well as test methods to manage missing baseline measurements (i.e., multiple imputation) for use in conjunction with CRS estimation. Another consideration was that in the analyses of the association of conditional velocity with stunting at age 2 years, the more complex approach (CRS) was not necessarily more informative than the more straightforward conditional SDS approach. This may have partly resulted from the artificial data structure we implemented (i.e., all children in the dataset had baseline and follow‐up LAZ) and the assumption of linearity, which may have minimized variability in velocity among children. Further research on the association between conditional velocity and later health outcomes is needed to weigh the advantages and disadvantages of using the CRS.

A limitation of this study was the lack of consideration of gestational age at birth, as this information was not available in both cohorts. Using chronological age for all children, irrespective of gestational age at birth, may bias the classification of growth trajectories, resulting in biased estimates of the association between growth velocity and stunting at 2 years (Perumal, Gaffey, Bassani, & Roth, 2015). Another weakness of the study was that our analyses were only applied to LAZ from 0 to 12 months. Further analyses are needed to demonstrate that the principles explored in this article are applicable to other anthropometric variables and in other age intervals of interest. Finally, we provided only a limited empiric demonstration of the association between LAZ velocity in the first year of life and a later outcome, to highlight the similarity between CRS and classical conditional SDS in an epidemiologic context, and to reinforce the divergent inferences that may be generated by conditional and unconditional models (Tu, Tilling, Sterne, & Gilthorpe, 2013). More comprehensive comparisons of the performance of the various growth modeling approaches for a range of health outcomes were beyond the scope of the present study.

Growth velocity in infancy is a key developmental indicator that is associated with long‐term outcomes (Adair et al., 2013; Brands, Demmelmair, & Koletzko, 2014; Kelishadi and Poursafa, 2014; Langley‐Evans, 2015; Stein et al., 2010). The CRS approach enables estimation of individual‐specific conditional growth velocities in longitudinal cohort studies in which there are multiple size measurements per child within the age interval of interest. Further efforts are required to extend and validate the applicability of the CRS approach to epidemiological studies in which researchers wish to investigate risk factors for relatively slow or fast child growth, or studies of association between early growth and later outcomes.

## ACKNOWLEDGMENTS

The authors thank the colleagues in the Healthy Birth, Growth, and Development Knowledge integration (HBGDki) consortium, and HBGDki staff at the Bill and Melinda Gates Foundation. They also acknowledge Wellcome Trust and the Bill and Melinda Gates Foundation for supporting the original cohort studies. They also thank the AJHB reviewer for valuable feedback on a previous version of this article.

## AUTHOR CONTRIBUTIONS

ML, DGB, AR, AG, and DER designed the study, developed the methodology, performed the analyses and wrote the manuscript. SAA, GK, and PSP collected the data. Each author has seen and approved the contents of the submitted manuscript.

## Supporting information


**Supporting Information**
