# Supplementary material for: Conditional random slope: A new approach for estimating individual child growth velocity in epidemiological research
Source: Am J Hum Biol. 2017 Apr 21;29(5):e23009. doi: 10.1002/ajhb.23009 (PMC5599979; doi:10.1002/ajhb.23009)
Supplement: Supplementary file 1 — Supporting Information [file AJHB-29-na-s001.docx]

**SUPPLEMENTARY MATERIAL**

Table of Contents

A. Sampling of observations 3

Table A1. Age at observation for data densities of 2-5 observations per child in Cohorts A and B 3

B. Derivation of the classical conditional standard deviation score (SDS) from the conditional random slope model 4

C. Population-average length-for-age z-scores at baseline and slopes from birth to 12 months of age in Cohorts A and B 5

Table C1. Comparison of fixed effect estimates of group mean LAZ slopes and standard errors (SE) from birth to 12 months and mean baseline LAZ using different analytical approaches and varying number of observations per child. 5

D. Linearity of population-average trajectories in Cohorts A and B 6

Figure D1. Individual LAZ trajectories (A) and population-average trajectories using linear and adaptive fits (B). Adaptive fits consisted of refitting the models using age bins with dummy variables. 6

E. Correlation, kappa and % discordance matrices of velocity metrics based on length-for-age z-scores from 0 to 365 days of age 7

Table E1. Correlation, kappa and % discordance matrix among growth velocity metrics for 2 observations per child^a,b^ 7

Table E2. Correlation, kappa and % discordance matrices among growth velocity metrics for 3, 4 and 5 observations per child^a,b^ 8

Table E3. Correlation, kappa and % discordance matrix among growth velocity metrics for 6 to 13 observations per child^a,b^ 9

F. Assessment of model fit using AIC and BIC 10

Table F1. Comparisons of Akaike information criterion (AIC) and Bayesian information criterion (BIC) among mixed-effects models used to estimate growth velocity with variable numbers of observations per child, in Tables 2-4 of the manuscript 10

G. Sensitivity Analysis #1: Unconditional random slope estimation using unstructured covariance 11

Table G1. Correlation between estimated change in length-for-age z-scores (LAZ) from birth to 12 months of age (velocity) and baseline LAZ using different analytical approaches and variable number of observations per child using an unstructured covariance matrix for the unconditional random slope model 11

Table G2. Pairwise comparisons using Pearson’s correlation (R), Kappa coefficient ($\kappa$) and percent discordance (%D) between conditional slopes and alternative metrics of growth velocity from birth to 12 months of age, by number of observations per child using an unstructured covariance matrix for the unconditional random slope model 12

Table G3. Comparison of the strength of association between stunting at 2 years of age and internally standardized estimates of growth velocity, using different analytical approaches and varying number of observations per child using an unstructured covariance matrix for the unconditional random slope model 13

H. Sensitivity Analysis #2: Conditional random slope with main effect for baseline size (LAZ_0_) included in models 14

Table H1. Correlation between estimated change in length-for-age z-scores (LAZ) from birth to 12 months of age (velocity) and baseline LAZ using different analytical approaches and variable number of observations per child including baseline as a main effect in the conditional random slope model 14

Table H2. Pairwise comparisons using Pearson’s correlation (R), Kappa coefficient ($\kappa$) and percent discordance (%D) between conditional random slopes and alternative metrics of growth velocity from birth to 12 months of age, by number of observations per child including baseline as a main effect in the conditional random slope model 15

Table H3. Comparison of the strength of association between stunting at 2 years of age and internally standardized estimates of growth velocity, using different analytical approaches and varying number of observations per child including baseline as a main effect in the conditional random slope model 16

I. Sensitivity Analysis #3: Exclusion of children with a baseline measurement at >1 week of age 17

Table I1. Correlation between estimated change in length-for-age z-scores (LAZ) from birth to 12 months of age (velocity) and baseline LAZ using different analytical approaches and variable number of observations per child excluding children with a baseline measurement beyond the first week of life 17

Table I2. Pairwise comparisons using Pearson’s correlation (R), Kappa coefficient ($\kappa$) and percent discordance (%D) between conditional slopes and alternative metrics of growth velocity from birth to 12 months of age, by number of observations per child excluding children with a baseline measurement beyond the first week of life 18

Table I3. Comparison of the strength of association between stunting at 2 years of age and internally standardized estimates of growth velocity, using different analytical approaches and varying number of observations per child excluding children with a baseline measurement beyond the first week of life 19

# A. Sampling of observations

## Table A1. Age at observation for data densities of 2-5 observations per child in Cohorts A and B

| **# of observations per child^a^** | Age in days at observation^b^, median (range) | |
| --- | --- | --- |
| Observation number | Cohort A | Cohort B |
| **2 Observations per child** | | |
| 1 | 29 (1, 66) | 7 (1, 68) |
| 2 | 347 (316, 365) | 340 (306, 355) |
| **3 Observations per child** | | |
| 1 | 29 (1, 66) | 7 (1, 68) |
| 2 | 179 (123, 253) | 188 (154, 251) |
| 3 | 347 (316, 365) | 340 (306, 355) |
| **4 Observations per child** | | |
| 1 | 29 (1, 66) | 7 (1, 68) |
| 2 | 116 (71, 158) | 127 (63, 161) |
| 3 | 244 (185, 309) | 249 (196, 277) |
| 4 | 347 (316, 365) | 340 (306, 355) |
| **5 Observations per child** | | |
| 1 | 29 (1, 66) | 7 (1, 68) |
| 2 | 86 (49, 120) | 96 (63, 125) |
| 3 | 179 (123, 253) | 188 (154, 251) |
| 4 | 277 (244, 309) | 279 (226, 317) |
| 5 | 347 (316, 365) | 340 (306, 355) |

^a^ Every child has an observation at or close to birth and at or close to 12 months of age, and up to 3 additional observations included within the interval from birth to 12 months of age.

^b^ Day 1 is day of birth

# B. Derivation of the classical conditional standard deviation score (SDS) from the conditional random slope model

LAZ_ij_ = $\beta$_0_ + $\beta$_1_(t) + $\mu$_0j_+ $\mu$_1j_(t) + $\beta$_2_(t*LAZ_0j_) + $\varepsilon$_ij_ (1)

Expected baseline is defined by the fixed and random intercepts:

E(LAZ_0j_) = $\beta$_0_ + $\mu$_0j_  (2)

Sub (2) into (1)

LAZ_ij_ = E(LAZ_0j_) + $\beta$_1_(t) + $\mu$_1j_(t) + $\beta$_2_(t*LAZ_0j_) + $\varepsilon$_ij_ (3)

Conditional expectation of LAZ_ij_ is defined by the sum of baseline size, fixed slope and baseline-time interaction:

E_c_(LAZ_ij_) = E(LAZ_0j_) + $\beta$_1_(t) + $\beta$_2_(t*LAZ_0j_) + $\varepsilon$_ij_ (4)

Sub (4) into (3)

LAZ_ij_ = E_c_(LAZ_ij_) + $\mu$_1j_(t) + $\varepsilon$_ij_ (5)

Re-arrange (5)

$\mu$_1j_(t) = LAZ_ij_ - E_c_(LAZ_ij_) + $\varepsilon$_ij_ (6)

If we restrict (6) to the conditions used to estimate conditional SDS (only two points are observed for every child, one at the beginning and the end of the interval) and within-child variation/error is assumed to be 0, then t (duration of the interval) is constant across all children and $\varepsilon$_ij_=0.

$\mu$_1j_ = LAZ_1j_ - E_c_(LAZ_1j_) (7)

$\therefore\mu$_1j_ is equivalent to the conditional SDS, in that they both represent the difference between observed size of the child at the end of the interval and the child’s expected size, which is conditioned by his or her size at a preceding age.

# C. Population-average length-for-age z-scores at baseline and slopes from birth to 12 months of age in Cohorts A and B

## **Table C1**. Comparison of fixed effect estimates of group mean LAZ slopes and standard errors (SE) from birth to 12 months and mean baseline LAZ using different analytical approaches and varying number of observations per child.

| **Cohort** | Unconditional Delta | | Fixed Slope^a^ | | Random Slope^b^ | | Conditional Random Slope^b^ | |
| --- | --- | --- | --- | --- | --- | --- | --- | --- |
| # of observations per child^c^ | Mean Slope^d^ (SE) | Mean LAZ at age=0 (SE) | Mean Slope^d^ (SE) | Mean LAZ at age=0 (SE) | Mean Slope^d^ (SE) | Mean LAZ at age=0 (SE) | Mean Slope^d^ (SE) | Mean LAZ at age=0 (SE) |
| **Cohort A** | | | | | | | | |
| 2 | -1.03 (0.08) | -0.94 (0.07) | -1.18 (0.09) | -0.84 (0.08) | -1.15 (0.09) | -0.87 (0.06) | -1.15 (0.08) | -0.89 (0.06) |
| 3 | - | - | -1.17 (0.09) | -0.87 (0.07) | -1.15 (0.09) | -0.89 (0.06) | -1.14 (0.08) | -0.89 (0.06) |
| 4 | - | - | -1.12 (0.09) | -0.89 (0.07) | -1.11 (0.09) | -1.00 (0.06) | -1.11 (0.08) | -1.00 (0.06) |
| 5 | - | - | -1.10 (0.09) | -0.90 (0.07) | -1.09 (0.09) | -0.91 (0.06) | -1.08 (0.08) | -0.91 (0.06) |
| All | - | - | -1.05 (0.09) | -0.94 (0.07) | -1.05 (0.09) | -0.95 (0.06) | -1.05 (0.08) | -0.95 (0.06) |
| **Cohort B** | | | | | | | | |
| 2 | -1.22 (0.08) | -1.42 (0.09) | -1.33 (0.08) | -1.39 (0.09) | -1.37 (0.08) | -1.34 (0.06) | -1.34 (0.05) | -1.36 (0.06) |
| 3 | - | - | -1.34 (0.08) | -1.42 (0.08) | -1.35 (0.08) | -1.40 (0.06) | -1.35 (0.05) | -1.40 (0.06) |
| 4 | - | - | -1.14 (0.08) | -1.60 (0.08) | -1.14 (0.08) | -1.69 (0.06) | -1.14 (0.05) | -1.69 (0.06) |
| 5 | - | - | -1.02 (0.08) | -1.68 (0.08) | -1.03 (0.08) | -1.68 (0.06) | -1.02 (0.06) | -1.68 (0.06) |
| All | - | - | -0.60 (0.08) | -1.97 (0.08) | -0.61 (0.08) | -1.95 (0.06) | -0.60 (0.06) | -1.96 (0.06) |

^a^ Mean LAZ at age=0 is the intercept

^b^ Age was centered at its mean; therefore, mean LAZ at age=0 was generated by extrapolating the mean slope to age=0

^c^ Every child has at least five observations: at or close to birth, at or close to 12 months of age, and up to 3 additional observations included within the interval from birth to 12 months of age. Analyses of ‘all’ observations included the core five and all other available observations to a maximum of 13 observations per child.

^d^ Units: LAZ/year

# D. Linearity of population-average trajectories in Cohorts A and B

**Cohort A**

(B)

(A)


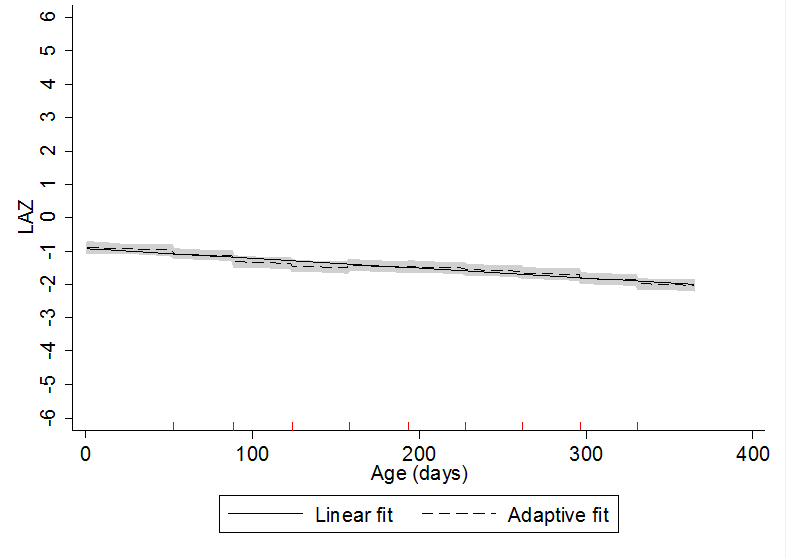


**Cohort B**

(B)

(A)

**
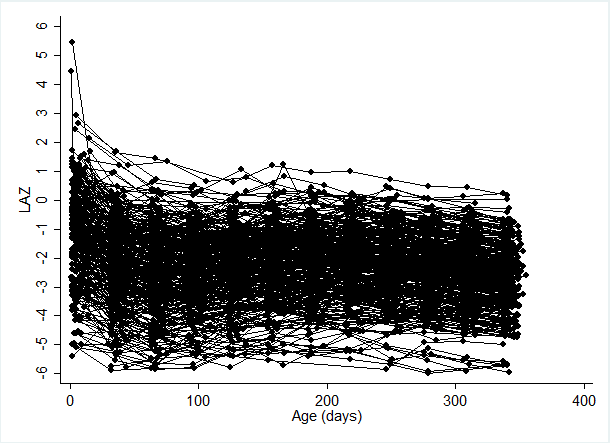

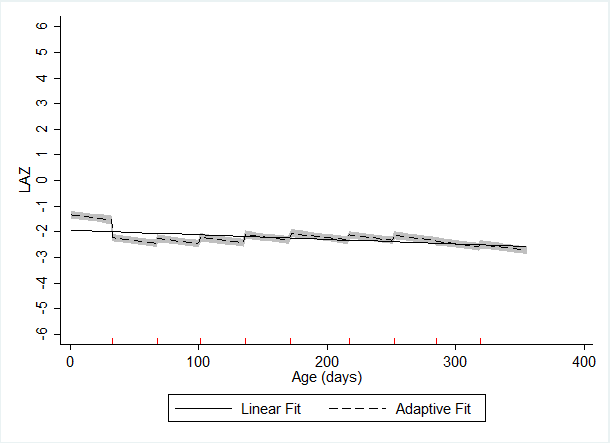
**

## Figure D1. Individual LAZ trajectories (A) and population-average trajectories using linear and adaptive fits (B). Adaptive fits consisted of refitting the models using age bins with dummy variables.

# E. Correlation, kappa and % discordance matrices of velocity metrics based on length-for-age z-scores from 0 to 365 days of age

## **Table E1**. Correlation, kappa and % discordance matrix among growth velocity metrics for 2 observations per child^a,b^

| Metric | Cohort A (n=348) | | | | | Cohort B (n=362) | | | | |
| --- | --- | --- | --- | --- | --- | --- | --- | --- | --- | --- |
|  | c$\Delta$LAZ | $\Delta$LAZ | Fixed | Random | CRS | c$\Delta$LAZ | $\Delta$LAZ | Fixed | Random | CRS |
| **2 Observations per child** | | | | | | | | | | |
| c$\Delta$LAZ | 1.00^c^  1.00^d^  0.0%^d^ | - | - | - | - | 1.00  1.00  0.0% | - | - | - | - |
| $\Delta$LAZ | 0.82  0.65  6.3% | 1.00  1.00  0.0% | - | - | - | 0.65  0.44  9.9% | 1.00  1.00  0.0% | - | - | - |
| Fixed | 0.82  0.68  5.8% | 0.99  0.90  1.7% | 1.00  1.00  0.0% | - | - | 0.65  0.41  10.5% | 0.99  0.97  0.6% | 1.00  1.00  0.0% | - | - |
| Random | 0.82  0.65  6.3% | 0.99  1.00  0.0% | 0.99  0.90  1.7% | 1.00  1.00  0.0% | - | 0.65  0.44  9.9% | 0.99  1.00  0.0% | 0.99  0.97  0.6% | 1.00  1.00  0.0% | - |
| CRS | 0.99  0.97  0.6% | 0.82  0.62  6.9% | 0.82  0.65  6.3% | 0.82  0.62  6.9% | 1.00  1.00  0.0% | 0.99  1.00  0.0% | 0.65  0.44  9.9% | 0.65  0.41  10.5% | 0.65  0.44  9.9% | 1.00  1.00  0.0% |

^a^ All correlation and kappa coefficients were statistically significant (p<0.05).

^b^ Every child has an observation at or close to birth and at or close to 12 months of age.

^c^ Pearson correlation coefficient between 2-point metrics of growth velocity.

^d^ Kappa and % discordance with respect to the classification of children as abnormal – where abnormal for each metric was defined as velocity estimates below the metric-specific 10^th^ percentile.

## **Table E2**. Correlation, kappa and % discordance matrices among growth velocity metrics for 3, 4 and 5 observations per child^a,b^

| Metric | Cohort A (n=348) | | | Cohort B (n=362) | | |
| --- | --- | --- | --- | --- | --- | --- |
|  | Fixed | Random | CRS | Fixed | Random | CRS |
| **3 observations per child** | | | | | | |
| Fixed | 1.00^c^  1.00^d^  0.0%^d^ | - | - | 1.00  1.00  0.0% | - | - |
| Random | 0.99  0.90  1.7% | 1.00  1.00  0.0% | - | 0.99  1.00  0.0% | 1.00  1.00  0.0% | - |
| CRS | 0.82  0.65  6.3% | 0.82  0.65  6.3% | 1.00  1.00  0.0% | 0.65  0.38  11.1% | 0.65  0.38  11.1% | 1.00  1.00  0.0% |
| **4 observations per child** | | | | | | |
| Fixed | 1.00  1.00  0.0% | - | - | 1.00  1.00  0.0% | - | - |
| Random | 0.99  0.97  0.6% | 1.00  1.00  0.0% | - | 0.99  1.00  0.0% | 1.00  1.00  0.0% | - |
| CRS | 0.87  0.65  6.3% | 0.87  0.62  6.9% | 1.00  1.00  0.0% | 0.70  0.51  8.8% | 0.70  0.51  8.8% | 1.00  1.00  0.0% |
| **5 observations per child** | | | | | | |
| Fixed | 1.00  1.00  0.0% | - | - | 1.00  1.00  0.0% | - | - |
| Random | 0.99  1.00  0.0% | 1.00  1.00  0.0% | - | 0.99  1.00  0.0% | 1.00  1.00  0.0% | - |
| CRS | 0.88  0.78  4.0% | 0.88  0.78  4.0% | 1.00  1.00  0.0% | 0.70  0.41  10.5% | 0.70  0.41  10.5% | 1.00  1.00  0.0% |

^a^ All correlation and kappa coefficients were statistically significant (p<0.05).

^b^ Every child has an observation at or close to birth and at or close to 12 months of age, with up to three additional observations within this interval.

^c^ Pearson correlation coefficient between metrics of growth velocity.

^d^ Kappa and % discordance with respect to the classification of children as abnormal – where abnormal for each metric was defined as velocity estimates below the metric-specific 10^th^ percentile.

## **Table E3**. Correlation, kappa and % discordance matrix among growth velocity metrics for 6 to 13 observations per child^a,b^

| Metric | Cohort A (n=348) | | | Cohort B (n=362) | | |
| --- | --- | --- | --- | --- | --- | --- |
|  | Fixed | Random | CRS | Fixed | Random | CRS |
| **All observations** | | | | | | |
| Fixed | 1.00^c^  1.00^d^  0.0%^d^ | - | - | 1.00  1.00  0.0% | - | - |
| Random | 0.99  0.84  2.9% | 1.00  1.00  0.0% | - | 0.99  0.81  3.3% | 1.00  1.00  0.0% | - |
| CRS | 0.91  0.65  6.3% | 0.91  0.71  5.2% | 1.00  1.00  0.0% | 0.78  0.41  10.5% | 0.78  0.48  9.4% | 1.00  1.00  0.0% |

^a^ All correlation and kappa coefficients were statistically significant (p<0.05).

^b^ Every child has a minimum of 6 points and a maximum of 13 points, where two of these points are at or close to birth and at or close to 12 months of age.

^c^ Pearson correlation coefficient between metrics of growth velocity.

^d^ Kappa and % discordance with respect to the classification of children as abnormal – where abnormal for each metric was defined as velocity estimates below the metric-specific 10^th^ percentile.

# F. Assessment of model fit using AIC and BIC

## **Table F1**. Comparisons of Akaike information criterion (AIC) and Bayesian information criterion (BIC) among mixed-effects models used to estimate growth velocity with variable numbers of observations per child, in Tables 2-4 of the manuscript

| **Cohort** | Random Slope | | Conditional Random Slope | |
| --- | --- | --- | --- | --- |
| # of observations per child^a^ | AIC | BIC | AIC | BIC |
| **Cohort A** | | | | |
| 2 | 2336 | 2359 | 2198 | 2225 |
| 3 | 3410 | 3435 | 3275 | 3305 |
| 4 | 4988 | 5015 | 4894 | 4926 |
| 5 | 5244 | 5271 | 5161 | 5194 |
| All | 9876 | 9907 | 9813 | 9850 |
| **Cohort B** | | | | |
| 2 | 2469 | 2491 | 2165 | 2192 |
| 3 | 3283 | 3308 | 2974 | 3004 |
| 4 | 4649 | 4676 | 4395 | 4428 |
| 5 | 4912 | 4939 | 4657 | 4690 |
| All | 8872 | 8904 | 8698 | 8736 |

^a^ Every child has at least five observations: at or close to birth, at or close to 12 months of age, and up to 3 additional observations included within the interval from birth to 12 months of age. Analyses of ‘all’ observations included the core five and all other available observations to a maximum of 13 observations per child.

# G. Sensitivity Analysis #1: Unconditional random slope estimation using unstructured covariance

## **Table G1**. Correlation between estimated change in length-for-age z-scores (LAZ) from birth to 12 months of age (velocity) and baseline LAZ using different analytical approaches and variable number of observations per child using an unstructured covariance matrix for the unconditional random slope model

| **Cohort** | Velocity – Baseline Correlation^a^ | | | | |
| --- | --- | --- | --- | --- | --- |
| # of observations per child^b^ | Conditional Delta  vs. Baseline | Unconditional Delta  vs. Baseline | Fixed Slope  vs. Baseline | Random Slope  vs. Baseline | Conditional Random Slope  vs. Baseline |
| **Cohort A** | | | | | |
| 2 | 0.00 | -0.57 | -0.57 | -0.58 | 0.00 |
| 3 | - | - | -0.56 | -0.28 | 0.00 |
| 4 | - | - | -0.49 | -0.40 | 0.00 |
| 5 | - | - | -0.48 | -0.38 | 0.00 |
| All | - | - | -0.41 | -0.38 | 0.00 |
| **Cohort B** | | | | | |
| 2 | 0.00 | -0.76 | -0.76 | * | 0.00 |
| 3 | - | - | -0.76 | -0.88 | 0.00 |
| 4 | - | - | -0.71 | -0.75 | 0.00 |
| 5 | - | - | -0.71 | -0.78 | 0.00 |
| All | - | - | -0.62 | -0.64 | 0.00 |

^a^ All correlation coefficients for unconditional metrics were statistically significant (p<0.05).

^b^ Every child has at least five observations: at or close to birth, at or close to 12 months of age, and up to 3 additional observations included within the interval from birth to 12 months of age. Analyses of ‘all’ observations included the core five and all other available observations to a maximum of 13 observations per child.

*Did not converge

## **Table G2**. Pairwise comparisons using Pearson’s correlation (R), Kappa coefficient ($\boldsymbol{\kappa}$) and percent discordance (%D) between conditional slopes and alternative metrics of growth velocity from birth to 12 months of age, by number of observations per child using an unstructured covariance matrix for the unconditional random slope model

| **Cohort** | Conditional Delta vs.  Conditional Random Slope | | | Unconditional Delta vs.  Conditional Random Slope | | | Fixed Slope vs.  Conditional Random Slope | | | Random Slope vs.  Conditional Random Slope | | |
| --- | --- | --- | --- | --- | --- | --- | --- | --- | --- | --- | --- | --- |
| # of observations per child^a^ | R^b^ | $\kappa$^b,c^ | %D^b,c^ | R^b^ | $\kappa$^b,c^ | %D^b,c^ | R^b^ | $\kappa$^b,c^ | %D^b,c^ | R^b^ | $\kappa$^b,c^ | %D^b,c^ |
| **Cohort A** | | | | | | | | | | | | |
| 2 | 0.99 | 0.97 | 0.6% | 0.82 | 0.62 | 6.9% | 0.82 | 0.65 | 6.3% | 0.81 | 0.62 | 6.9% |
| 3 | - | - | - | - | - | - | 0.82 | 0.65 | 6.3% | 0.95 | 0.84 | 2.9% |
| 4 | - | - | - | - | - | - | 0.87 | 0.65 | 6.3% | 0.92 | 0.75 | 4.6% |
| 5 | - | - | - | - | - | - | 0.88 | 0.78 | 4.0% | 0.92 | 0.87 | 2.3% |
| All | - | - | - | - | - | - | 0.91 | 0.65 | 6.3% | 0.92 | 0.68 | 5.8% |
| **Cohort B** | | | | | | | | | | | | |
| 2 | 0.99 | 1.00 | 0.0% | 0.65 | 0.44 | 9.9% | 0.65 | 0.41 | 10.5% | * | * | * |
| 3 | - | - | - | - | - | - | 0.65 | 0.38 | 11.0% | 0.47 | 0.23 | 13.8% |
| 4 | - | - | - | - | - | - | 0.70 | 0.51 | 8.8% | 0.66 | 0.32 | 12.2% |
| 5 | - | - | - | - | - | - | 0.70 | 0.41 | 10.5% | 0.61 | 0.44 | 9.9% |
| All | - | - | - | - | - | - | 0.78 | 0.41 | 10.5% | 0.77 | 0.51 | 8.8% |

^a^ Every child has at least five observations: at or close to birth, at or close to 12 months of age, and up to 3 additional observations included within the interval from birth to 12 months of age. Analyses of ‘all’ observations included the core five and all other available observations to a maximum of 13 observations per child.

^b^ All correlation and kappa coefficients were statistically significant (p<0.05).

^c^ Kappa and % discordance with respect to the classification of children as ‘abnormal’ - where abnormal for each metric was defined as velocity estimates below the metric-specific 10^th^ percentile.

*Did not converge

## **Table G3**. Comparison of the strength of association between stunting at 2 years of age and internally standardized estimates of growth velocity, using different analytical approaches and varying number of observations per child using an unstructured covariance matrix for the unconditional random slope model

| **Cohort** | Conditional Delta | | Unconditional Delta | | Fixed Slope | | Random Slope | | Conditional Random Slope | |
| --- | --- | --- | --- | --- | --- | --- | --- | --- | --- | --- |
| # of observations per child^a^ | OR^b^ (95% CI) | C^c^ | OR^b^ (95% CI) | C^c^ | OR^b^ (95% CI) | C^c^ | OR^b^ (95% CI) | C^c^ | OR^b^ (95% CI) | C^c^ |
| **Cohort A** | | | | | | | | | | |
| 2 | 2.19 (1.64, 2.91) | 0.71 | 1.46 (1.14, 1.88) | 0.61 | 1.47 (1.14, 1.89) | 0.61 | 1.44 (1.12, 1.84) | 0.60 | 2.11 (1.60, 2.80) | 0.71 |
| 3 | - | - | - | - | 1.47 (1.14, 1.89) | 0.61 | 1.87 (1.43, 2.44) | 0.68 | 2.11 (1.59, 2.79) | 0.71 |
| 4 | - | - | - | - | 1.46 (1.14, 1.88) | 0.60 | 1.60 (1.24, 2.07) | 0.63 | 1.95 (1.48, 2.57) | 0.68 |
| 5 | - | - | - | - | 1.56 (1.22, 2.02) | 0.62 | 1.71 (1.32, 2.21) | 0.65 | 2.10 (1.59, 2.78) | 0.70 |
| All | - | - | - | - | 1.58 (1.23, 2.03) | 0.63 | 1.62 (1.26, 2.09) | 0.64 | 2.00 (1.52, 2.63) | 0.69 |
| **Cohort B** | | | | | | | | | | |
| 2 | 8.76 (4.90, 15.69) | 0.89 | 1.30 (0.96, 1.76) | 0.57 | 1.30 (0.96, 1.75) | 0.57 | * | * | 9.03 (5.01, 16.3) | 0.89 |
| 3 | - | - | - | - | 1.30 (0.96, 1.75) | 0.57 | 0.93 (0.70, 1.25) | 0.53 | 9.39 (5.16, 17.07) | 0.90 |
| 4 | - | - | - | - | 1.37 (1.02, 1.84) | 0.59 | 1.25 (0.93, 1.69) | 0.56 | 6.10 (3.74, 9.95) | 0.87 |
| 5 | - | - | - | - | 1.34 (0.99, 1.80) | 0.59 | 1.12 (0.83, 1.49) | 0.53 | 5.71 (3.55, 9.21) | 0.87 |
| All | - | - | - | - | 1.37 (1.02, 1.83) | 0.60 | 1.31 (0.98, 1.75) | 0.59 | 3.38 (2.32, 4.92) | 0.81 |

^a^ Every child has at least five observations: at or close to birth, at or close to 12 months of age, and up to 3 additional observations included within the interval from birth to 12 months of age. Analyses of ‘all’ observations included the core five and all other available observations to a maximum of 13 observations per child.

^b^ Odds of stunting at 2 years of age for every 1 SD decrease in internally standardized estimates of growth velocity

^c^ C-statistic: area under the receiving operating characteristic (ROC) curve

*Did not converge

# H. Sensitivity Analysis #2: Conditional random slope with main effect for baseline size (LAZ_0_) included in models

## **Table H1**. Correlation between estimated change in length-for-age z-scores (LAZ) from birth to 12 months of age (velocity) and baseline LAZ using different analytical approaches and variable number of observations per child including baseline as a main effect in the conditional random slope model

| **Cohort** | Velocity – Baseline Correlation^a^ | | | | |
| --- | --- | --- | --- | --- | --- |
| # of observations per child^b^ | Conditional Delta  vs. Baseline | Unconditional Delta  vs. Baseline | Fixed Slope  vs. Baseline | Random Slope  vs. Baseline | Conditional Random Slope  vs. Baseline |
| **Cohort A** | | | | | |
| 2 | 0.00 | -0.57 | -0.57 | -0.57 | 0.00 |
| 3 | - | - | -0.56 | -0.56 | 0.00 |
| 4 | - | - | -0.49 | -0.49 | 0.00 |
| 5 | - | - | -0.48 | -0.48 | 0.00 |
| All | - | - | -0.41 | -0.41 | 0.00 |
| **Cohort B** | | | | | |
| 2 | 0.00 | -0.76 | -0.76 | -0.76 | 0.00 |
| 3 | - | - | -0.76 | -0.76 | 0.00 |
| 4 | - | - | -0.71 | -0.71 | 0.00 |
| 5 | - | - | -0.71 | -0.71 | 0.00 |
| All | - | - | -0.62 | -0.62 | 0.00 |

^a^ All correlation coefficients for unconditional metrics were statistically significant (p<0.05).

^b^ Every child has at least five observations: at or close to birth, at or close to 12 months of age, and up to 3 additional observations included within the interval from birth to 12 months of age. Analyses of ‘all’ observations included the core five and all other available observations to a maximum of 13 observations per child.

## **Table H2**. Pairwise comparisons using Pearson’s correlation (R), Kappa coefficient ($\boldsymbol{\kappa}$) and percent discordance (%D) between conditional random slopes and alternative metrics of growth velocity from birth to 12 months of age, by number of observations per child including baseline as a main effect in the conditional random slope model

| **Cohort** | Conditional Delta vs.  Conditional Random Slope | | | Unconditional Delta vs.  Conditional Random Slope | | | Fixed Slope vs.  Conditional Random Slope | | | Random Slope vs.  Conditional Random Slope | | |
| --- | --- | --- | --- | --- | --- | --- | --- | --- | --- | --- | --- | --- |
| # of observations per child^a^ | R^b^ | $\kappa$^b,c^ | %D^b,c^ | R^b^ | $\kappa$^b,c^ | %D^b,c^ | R^b^ | $\kappa$^b,c^ | %D^b,c^ | R^b^ | $\kappa$^b,c^ | %D^b,c^ |
| **Cohort A** | | | | | | | | | | | | |
| 2 | 0.99 | 0.97 | 0.6% | 0.82 | 0.62 | 6.9% | 0.82 | 0.65 | 6.3% | 0.82 | 0.62 | 6.9% |
| 3 | - | - | - | - | - | - | 0.82 | 0.65 | 6.3% | 0.82 | 0.65 | 6.3% |
| 4 | - | - | - | - | - | - | 0.87 | 0.65 | 6.3% | 0.87 | 0.62 | 6.9% |
| 5 | - | - | - | - | - | - | 0.88 | 0.78 | 4.0% | 0.88 | 0.78 | 4.0% |
| All | - | - | - | - | - | - | 0.91 | 0.65 | 6.3% | 0.91 | 0.71 | 5.2% |
| **Cohort B** | | | | | | | | | | | | |
| 2 | 0.99 | 1.00 | 0.0% | 0.65 | 0.44 | 9.9% | 0.65 | 0.41 | 10.5% | 0.65 | 0.44 | 9.9% |
| 3 | - | - | - | - | - | - | 0.65 | 0.38 | 11.1% | 0.65 | 0.38 | 11.1% |
| 4 | - | - | - | - | - | - | 0.70 | 0.51 | 8.8% | 0.70 | 0.51 | 8.8% |
| 5 | - | - | - | - | - | - | 0.70 | 0.41 | 10.5% | 0.70 | 0.41 | 10.5% |
| All | - | - | - | - | - | - | 0.78 | 0.41 | 10.5% | 0.78 | 0.48 | 9.4% |

^a^ Every child has at least five observations: at or close to birth, at or close to 12 months of age, and up to 3 additional observations included within the interval from birth to 12 months of age. Analyses of ‘all’ observations included the core five and all other available observations to a maximum of 13 observations per child.

^b^ All correlation and kappa coefficients were statistically significant (p<0.05).

^c^ Kappa and % discordance with respect to the classification of children as ‘abnormal’ - where abnormal for each metric was defined as velocity estimates below the metric-specific 10^th^ percentile.

## **Table H3**. Comparison of the strength of association between stunting at 2 years of age and internally standardized estimates of growth velocity, using different analytical approaches and varying number of observations per child including baseline as a main effect in the conditional random slope model

| **Cohort** | Conditional Delta | | Unconditional Delta | | Fixed Slope | | Random Slope | | Conditional Random Slope | |
| --- | --- | --- | --- | --- | --- | --- | --- | --- | --- | --- |
| # of observations per child^a^ | OR^b^ (95% CI) | C^c^ | OR^b^ (95% CI) | C^c^ | OR^b^ (95% CI) | C^c^ | OR^b^ (95% CI) | C^c^ | OR^b^ (95% CI) | C^c^ |
| **Cohort A** | | | | | | | | | | |
| 2 | 2.19 (1.64, 2.91) | 0.71 | 1.46 (1.14, 1.88) | 0.61 | 1.47 (1.14, 1.89) | 0.61 | 1.45 (1.13, 1.86) | 0.61 | 2.11 (1.59, 2.79) | 0.71 |
| 3 | - | - | - | - | 1.47 (1.14, 1.89) | 0.61 | 1.45 (1.13, 1.87) | 0.61 | 2.11 (1.59, 2.79) | 0.71 |
| 4 | - | - | - | - | 1.46 (1.14, 1.88) | 0.60 | 1.46 (1.14, 1.87) | 0.60 | 1.95 (1.48, 2.57) | 0.68 |
| 5 | - | - | - | - | 1.56 (1.22, 2.02) | 0.62 | 1.56 (1.21, 2.00) | 0.62 | 2.10 (1.59, 2.78) | 0.70 |
| All | - | - | - | - | 1.58 (1.23, 2.03) | 0.63 | 1.57 (1.22, 2.02) | 0.63 | 2.00 (1.52, 2.63) | 0.69 |
| **Cohort B** | | | | | | | | | | |
| 2 | 8.76 (4.90, 15.69) | 0.89 | 1.30 (0.96, 1.76) | 0.57 | 1.30 (0.96, 1.75) | 0.57 | 1.32 (0.97, 1.79) | 0.58 | 9.03 (5.01, 16.3) | 0.89 |
| 3 | - | - | - | - | 1.30 (0.96, 1.75) | 0.57 | 1.31 (0.97, 1.77) | 0.57 | 9.39 (5.16, 17.1) | 0.90 |
| 4 | - | - | - | - | 1.37 (1.02, 1.84) | 0.59 | 1.38 (1.02, 1.85) | 0.59 | 6.10 (3.74, 9.95) | 0.87 |
| 5 | - | - | - | - | 1.34 (0.99, 1.80) | 0.59 | 1.35 (1.00, 1.81) | 0.59 | 5.71 (3.55, 9.21) | 0.87 |
| All | - | - | - | - | 1.37 (1.02, 1.83) | 0.60 | 1.38 (1.03, 1.85) | 0.60 | 3.38 (2.32, 4.92) | 0.81 |

^a^ Every child has at least five observations: at or close to birth, at or close to 12 months of age, and up to 3 additional observations included within the interval from birth to 12 months of age. Analyses of ‘all’ observation included the core five and all other available observations to a maximum of 13 observations per child.

^b^ Odds of stunting at 2 years of age for every 1 SD decrease in internally standardized estimates of growth velocity

^c^ C-statistic: area under the receiving operating characteristic (ROC) curve

# I. Sensitivity Analysis #3: Exclusion of children with a baseline measurement at >1 week of age

## **Table I1**. Correlation between estimated change in length-for-age z-scores (LAZ) from birth to 12 months of age (velocity) and baseline LAZ using different analytical approaches and variable number of observations per child excluding children with a baseline measurement beyond the first week of life

| **Cohort** | Velocity – Baseline Correlation^a^ | | | | |
| --- | --- | --- | --- | --- | --- |
| # of observations per child^b^ | Conditional Delta  vs. Baseline | Unconditional Delta  vs. Baseline | Fixed Slope  vs. Baseline | Random Slope  vs. Baseline | Conditional Random Slope  vs. Baseline |
| **Cohort A (n=88)** | | | | | |
| 2 | 0.00 | -0.55 | -0.55 | -0.55 | 0.00 |
| 3 | - | - | -0.55 | -0.56 | 0.00 |
| 4 | - | - | -0.50 | -0.50 | 0.00 |
| 5 | - | - | -0.49 | -0.49 | 0.00 |
| All | - | - | -0.36 | -0.36 | 0.00 |
| **Cohort B (n=196)** | | | | | |
| 2 | 0.00 | -0.74 | -0.73 | -0.73 | 0.00 |
| 3 | - | - | -0.74 | -0.74 | 0.00 |
| 4 | - | - | -0.68 | -0.68 | 0.00 |
| 5 | - | - | -0.68 | -0.68 | 0.00 |
| All | - | - | -0.56 | -0.56 | 0.00 |

^a^ All correlation coefficients for unconditional metrics were statistically significant (p<0.05).

^b^ Every child has at least five observations: at or close to birth, at or close to 12 months of age, and up to 3 additional observations included within the interval from birth to 12 months of age. Analyses of ‘all’ observations included the core five and all other available observations to a maximum of 13 observations per child.

## **Table I2**. Pairwise comparisons using Pearson’s correlation (R), Kappa coefficient ($\boldsymbol{\kappa}$) and percent discordance (%D) between conditional slopes and alternative metrics of growth velocity from birth to 12 months of age, by number of observations per child excluding children with a baseline measurement beyond the first week of life

| **Cohort** | Conditional Delta vs.  Conditional Random Slope | | | Unconditional Delta vs.  Conditional Random Slope | | | Fixed Slope vs.  Conditional Random Slope | | | Random Slope vs.  Conditional Random Slope | | |
| --- | --- | --- | --- | --- | --- | --- | --- | --- | --- | --- | --- | --- |
| # of observations per child^a^ | R^b^ | $\kappa$^b,c^ | %D^b,c^ | R^b^ | $\kappa$^b,c^ | %D^b,c^ | R^b^ | $\kappa$^b,c^ | %D^b,c^ | R^b^ | $\kappa$^b,c^ | %D^b,c^ |
| **Cohort A (n=88)** | | | | | | | | | | | | |
| 2 | 0.99 | 1.00 | 0.0% | 0.84 | 0.50 | 9.1% | 0.83 | 0.50 | 9.1% | 0.83 | 0.63 | 6.8% |
| 3 | - | - | - | - | - | - | 0.83 | 0.50 | 9.1% | 0.83 | 0.63 | 6.8% |
| 4 | - | - | - | - | - | - | 0.86 | 0.63 | 6.8% | 0.86 | 0.63 | 6.8% |
| 5 | - | - | - | - | - | - | 0.93 | 0.50 | 9.1% | 0.87 | 0.63 | 6.8% |
| All | - | - | - | - | - | - | 0.83 | 0.26 | 13.6% | 0.93 | 0.50 | 9.1% |
| **Cohort B (n=196)** | | | | | | | | | | | | |
| 2 | 0.99 | 1.00 | 0.0% | 0.68 | 0.44 | 10.2% | 0.68 | 0.39 | 11.2% | 0.68 | 0.39 | 11.2% |
| 3 | - | - | - | - | - | - | 0.68 | 0.39 | 11.2% | 0.68 | 0.44 | 10.2% |
| 4 | - | - | - | - | - | - | 0.73 | 0.50 | 9.2% | 0.73 | 0.44 | 10.2% |
| 5 | - | - | - | - | - | - | 0.74 | 0.44 | 10.2% | 0.74 | 0.39 | 11.2% |
| All | - | - | - | - | - | - | 0.83 | 0.50 | 9.2% | 0.83 | 0.55 | 8.2% |

^a^ Every child has at least five observations: at or close to birth, at or close to 12 months of age, and up to 3 additional observations included within the interval from birth to 12 months of age. Analyses of ‘all’ observations included the core five and all other available observations to a maximum of 13 observations per child.

^b^ All correlation and kappa coefficients were statistically significant (p<0.05).

^c^ Kappa and % discordance with respect to the classification of children as ‘abnormal’ - where abnormal for each metric was defined as velocity estimates below the metric-specific 10^th^ percentile.

## **Table I3**. Comparison of the strength of association between stunting at 2 years of age and internally standardized estimates of growth velocity, using different analytical approaches and varying number of observations per child excluding children with a baseline measurement beyond the first week of life

| **Cohort** | Conditional delta | | Unconditional delta | | Fixed slope | | Random slope | | CRS | |
| --- | --- | --- | --- | --- | --- | --- | --- | --- | --- | --- |
| # of observations per child^a^ | OR^b^ (95% CI) | C^c^ | OR^b^ (95% CI) | C^c^ | OR^b^ (95% CI) | C^c^ | OR^b^ (95% CI) | C^c^ | OR^b^ (95% CI) | C^c^ |
| **Cohort A (n=88)** | | | | | | | | | | |
| 2 | 1.73 (1.03, 2.92) | 0.63 | 1.47 (0.88, 2.45) | 0.61 | 1.48 (0.88, 2.47) | 0.62 | 1.46 (0.87, 2.43) | 0.61 | 1.73 (1.03, 2.92) | 0.64 |
| 3 | - | - | - | - | 1.45 (0.87, 2.43) | 0.61 | 1.44 (0.86, 2.40) | 0.61 | 1.71 (1.02, 2.88) | 0.64 |
| 4 | - | - | - | - | 1.53 (0.90, 2.57) | 0.62 | 1.52 (0.90, 2.57) | 0.62 | 1.79 (1.04, 3.06) | 0.64 |
| 5 | - | - | - | - | 1.56 (0.93, 2.69) | 0.63 | 1.57 (0.93, 2.64) | 0.63 | 1.83 (1.07, 3.11) | 0.65 |
| All | - | - | - | - | 1.77 (1.05, 2.97) | 0.68 | 1.77 (1.05, 3.00) | 0.69 | 2.01 (1.16, 3.47) | 0.70 |
| **Cohort B (n=196)** | | | | | | | | | | |
| 2 | 8.12 (3.95, 16.7) | 0.90 | 1.50 (0.99, 2.25) | 0.63 | 1.49 (0.99, 2.24) | 0.63 | 1.49 (0.99, 2.24) | 0.63 | 7.82 (3.85, 15.9) | 0.90 |
| 3 | - | - | - | - | 1.49 (0.99, 2.24) | 0.63 | 1.49 (0.99, 2.24) | 0.63 | 8.11 (3.94, 16.7) | 0.90 |
| 4 | - | - | - | - | 1.55 (1.03, 2.35) | 0.63 | 1.56 (1.03, 2.35) | 0.63 | 5.88 (3.11, 11.1) | 0.87 |
| 5 | - | - | - | - | 1.54 (1.03, 2.32) | 0.63 | 1.54 (1.02, 2.32) | 0.63 | 5.55 (2.99, 10.3) | 0.87 |
| All | - | - | - | - | 1.50 (1.00, 2.23) | 0.63 | 1.50 (1.01, 2.23) | 0.63 | 3.14 (1.92, 5.14) | 0.79 |

^a^ Every child has at least five observations: at or close to birth, at or close to 12 months of age, and up to 3 additional observations included within the interval from birth to 12 months of age. Analyses of ‘all’ observations included the core five and all other available observations to a maximum of 13 observations per child.

^b^ Odds of stunting at 2 years of age for every 1 SD decrease in internally standardized estimates of growth velocity

^c^ C-statistic: area under the receiving operating characteristic (ROC) curve
